# Emergence of core–peripheries in networks

**DOI:** 10.1038/ncomms10441

**Published:** 2016-01-29

**Authors:** T. Verma, F. Russmann, N.A.M. Araújo, J. Nagler, H.J. Herrmann

**Affiliations:** 1 ETH Zürich, Computational Physics for Engineering Materials, Institute for Building Materials, Wolfgang-Pauli-Strasse 27, HIT, CH-8093 Zürich, Switzerland; 2 Departamento de Física, Faculdade de Ciências, Universidade de Lisboa, P-1749-016 Lisboa, Portugal; 3 Centro de Física Teórica e Computacional, Universidade de Lisboa, 1749-016 Lisboa, Portugal; 4 Departamento de Física, Universidade Federal do Ceará, Campus do Pici, Ceará, Fortaleza 60455-760, Brazil

## Abstract

A number of important transport networks, such as the airline and trade networks of the world, exhibit a characteristic core–periphery structure, wherein a few nodes are highly interconnected and the rest of the network frays into a tree. Mechanisms underlying the emergence of core–peripheries, however, remain elusive. Here, we demonstrate that a simple pruning process based on removal of underutilized links and redistribution of loads can lead to the emergence of core–peripheries. Links are assumed beneficial if they either carry a sufficiently large load or are essential for global connectivity. This incentivized redistribution process is controlled by a single parameter, which balances connectivity and profit. The obtained networks exhibit a highly resilient and connected core with a frayed periphery. The balanced network shows a higher resilience than the world airline network or the world trade network, revealing a pathway towards robust structural features through pruning.

## Abstract (web summary)

Many transport networks exhibit a core–periphery structure, where few nodes are highly interconnected and the rest form a tree-shaped structure. Here, the authors demonstrate how to generate such a structure through a pruning process based on removal of underutilized links and redistribution of loads.

## Graphical abstract

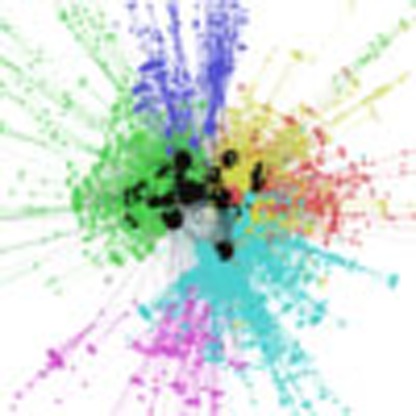

In today's world we want to fly everywhere. Despite higher fuel prices and a wider consciousness for reducing carbon emissions, airplane travel is on the rise globally and is predicted to grow even further in the future^1^. Events like the shutdown of the entire European airspace, due to the eruption of the Icelandic volcano, Eyjafjallajökull, have demonstrated the importance of efficiency and reliability of the airline traffic^2^ and other transport networks, be it trade, Internet or trains.

An ideal point-to-point network topology would ensure the fastest transfer of loads in a transport network. However, the real-world imposes costs on transport networks and their actual structure is a result of a complex interplay of (among other factors) economic considerations of involved parties as well as political ties between different regions. For instance, most major airlines, nowadays, employ a hub-and-spoke philosophy, in which passengers are routed through a few central airports, depending on the size of the airline's fleet. In recent years, however, especially low-cost airlines (for example, Ryanair in Europe) have rediscovered the point-to-point philosophy, providing non-stop flights wherever sufficient demand exists^3^. This results in a denser and more clustered network as opposed to a hub-and-spoke one.

One of the remarkable features of the world airline network (WAN) is its small core (consisting of ∼2.5% of the airports) that is almost fully connected and surrounded by a vast periphery that is nearly tree-like and connected to the core through many regional and national hubs^4^. This block arrangement is prominently known as the core–periphery (CP) structure^5^,^6^,^7^,^8^, which was also reported for other infrastructure networks, such as the world trade network^9^,^10^, the autonomous Internet network^10^ and the financial interbank lending markets^11^, where the fraction of peripheral nodes varies from 45 to 85%. Rombach *et al.*^12^ have also found similar structures for friendship, voting and collaboration networks and Avin *et al.*^13^ for other social networks.

The reason behind CPs is still unclear. Some transport network^14^,^15^ models have been based on a greedy optimization of a particular evaluation function of distance, cost or time. None of the above studies, however, could reproduce the CP structure.

We hypothesize that the CPs are a result of a naturally existing state of the dynamics of networks that are driven by a balance between functional connectivity and load-based profit. As an illustration of this hypothesis, commercial airlines will very likely cancel a direct link if the number of passengers does not compensate for the associated costs. Here, we start with a Utopian network where each node is connected to every other node. Underutilized links are pruned and the load of such links is redistributed to guarantee the load transfer between nodes. Through this pruning model, we demonstrate that CP structures can be obtained.

## Results

### Model

Generally, in transport networks, load is anything that needs to be transported from one place to another. We start with an ideal fully connected and undirected network, where the load pertaining to a pair of nodes can be transferred bidirectionally (a full description of the algorithm is given in Supplementary Methods^16^).

We represent the network using an adjacency matrix *A*_*ij*_(*N*, *V*) with *N* nodes and *V* links representing whether or not there exists a direct link between any pair of nodes. Our reference network contains *N*=1,000 nodes. Since we are interested in transport networks, we consider that a link is characterized by its load *l*_*ij*_, cost *c*_*ij*_ and physical length *d*_*ij*_ (Euclidean distance between nodes, in km, taken randomly from a Gaussian distribution, *μ*=8.369 × 10^3^; and *σ*=4.954 × 10^3^. The nodes are spread around a sphere of the size of the Earth—see Supplementary Fig. 1 and Supplementary Note 1).

We define the profit of a link connecting nodes *i* and *j* as


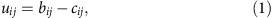


where *b*_*ij*_ is the benefit arising from a link and *c*_*ij*_ is the cost of establishing and maintaining the said link. Since the load of a link is a proxy for the benefit it accrues, we set *b*_*ij*_=*l*_*ij*_. For simplicity, we assign the same cost to every link with a dispersion to accommodate for heterogeneity in the network; *θ*≡*c*_*ij*_ and *c*_*ij*_=(1+*δ*_*ij*_)*c*, where *δ*_*ij*_ is a uniformly distributed random number in the range [−*a*; *a*]. In particular, we consider the cases *a*={0, 0.05, 0.1}. We obtain good quantitative agreement for the three cases, showing that our results are robust to heterogeneity in the parameter *c*_*ij*_ (see Supplementary Fig. 2 and Supplementary Note 2). Varying *θ* from the minimum load, we systematically prune links of negative profit, starting with the least loaded ones. An underutilized link that is necessary for maintaining global connectivity is not removed and classified as essential.

Once a link is pruned, its load is redistributed through the next best (shortest path) alternative, which potentially turns these alternative links more beneficial than they were before. In the case where several paths are of the same length, one is chosen at random. The load redistribution process can be explained in two steps. Firstly, when a link is removed, the load is routed through the next shortest path available between the nodes. Secondly, every link on the next available path will have to absorb the incoming load as it moves from source to sink. The reason for choosing the shortest path as the next available path is because normally in a transport network the length of travel times and in most passenger driven networks convenience is of primary importance to both the consumers and service providers. However, a robustness analysis of two other alternatives (random path and second shortest path) shows that CP features are observed in the critical window and the robustness of the networks in different regimes remains the same (see Supplementary Fig. 3). The pruning process eventually gives rise to a network only comprising essential links.

To distribute the loads, we introduce an observable called the popularity, *p*_*i*_, for each node *i*, characterizing its importance for the network. The popularity of a node is initially randomly chosen from a uniform distribution in the range [1/3,1], and alternatively from a scale-free distribution, *P*(*p*)∼*k*^−*γ*^, to contrast and compare the effect of initial conditions on our model (see Supplementary Fig. 4 and Supplementary Note 3). Subsequently, the initial load on any link is defined as the product of popularities of the nodes involved (see Supplementary Fig. 5, Supplementary Note 4 and Supplementary Movie for an in depth understanding of this relationship),


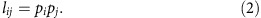


The popularity of each link remains intact with the pruning process. However, the load of each link dynamically changes as the load of removed links is redistributed. We have examined several other load functions (such as *l*_*ij*_=*p*_*i*_+*p*_*j*_, log(*p*_*i*_+*p*_*j*_), log(*p*_*i*_*p*_*j*_) and exp(*p*_*i*_*p*_*j*_)) and found no significant dependence of the main findings on the load function. In addition, we have also used a specific and more conventional case of load, betweenness centrality (see Supplementary Fig. 6 and Supplementary Note 5). As will be evident in the Results section, the existence of CPs remains the same. However, the load and its redistribution are needed (and critical) to find the CP structure.

**Figure 1:**
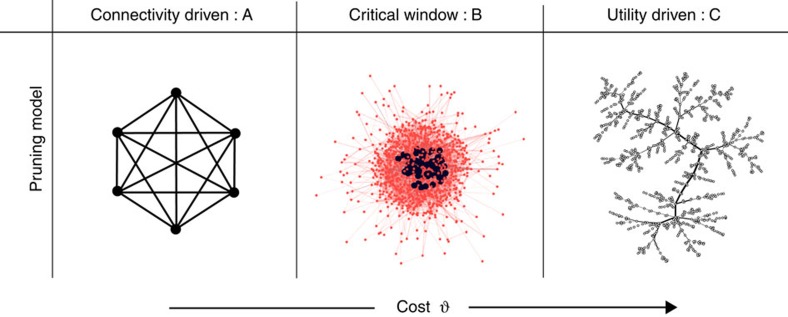
Schematic representation of the network classes obtained by our algorithm. For vanishing cost, the network is fully connected (network A of six nodes—shown for simplicity) resembling the initial network. For significantly high cost, the network is tree-like, exhibiting no loops (network C of 10^3^ nodes). In between, the proposed pruning process generates a network (network B of 10^3^ nodes) with a core–periphery structure. The network in regime B was obtained for cost, *θ*=0.92, corresponding to a peak in the core–periphery measure (details in the text). For the central network, the layout was generated by applying the Fruchterman–Reingold algorithm^23^. Colours show the difference in magnitude of coreness with black indicating the core and red, the periphery.

We run the above algorithm and analyse the structure using standard network techniques. The pruning process coupled with the load redistribution mechanism gives rise to three distinct families of network structures (see Fig. 1), one of which strongly resembles the features of a CP structure.

To identify and analyse the CP structure we use the *t*-core decomposition, as proposed in (ref. 4). Similar to the *k*-core decomposition^17^, this method progressively prunes a network by recursively removing nodes that are part of the least number of triangles. The decomposition assigns the removed nodes a ‘coreness', *t*, and places them in different shells, *t*=0, 1, 2…, where a shell, *t*, has nodes that are part of at least *t* triangles. Since triangles enhance the resilience of load transfer and this method recovers subgraphs at every shell that are more and more densely connected, the method uncovers a hierarchical ordering. More specifically, the load passing between a pair of nodes in a transport network can be redistributed with only one change in case the direct link becomes unavailable. A node that is part of the fully connected core of a network will be able to transfer its load through many alternatives (as many as there are nodes in the core) to accommodate for a faulty link. Thus, the *t*-core measure is especially suitable to assess which nodes belong to the core or the periphery.

To compare networks of different sizes, we define the relative coreness


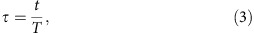


where 
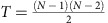
 is the maximum possible coreness of a node in a network of *N* nodes.

To perform a more aggregate level analysis where CP structure across different networks can be studied, we focus on the CP measure, a dimensionless quantity defined as


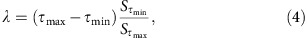


where 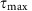 and 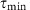 stand for the maximum and minimum relative coreness found in the network, respectively, and 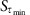 and 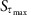 for the number of nodes that were assigned the respective coreness. A network with a genuine CP structure will have both, many nodes with low coreness (periphery) and a few nodes with high coreness (core). For example, the empirical WAN has a ratio, 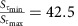, that is much larger than unity, suggesting the presence of very few nodes in the core, compared with the periphery. Thus, a high ratio indicates a particularly pronounced CP, and a low value, the lack of a CP. The rationale behind definition (4) is based on qualitative experience with the empirical WAN, which distinctly maximizes *λ* as there are very few nodes in the core and the majority of nodes fall in the periphery. Moreover, the difference in the relative coreness between core and periphery 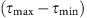 is large.

### Regimes

The cost, *θ*, is varied as an independent tunable parameter and the properties of the model networks are investigated as a function of this parameter. Specifically, we systematically increase the value of the cost, starting from the minimum load and until only essential links remain, namely links necessary to keep global connectivity.

Our pruning process, depending on the value of *θ*, necessarily leads to a crossover between different regimes of networks. Say that *l*_min_ and *l*_max_ are the least and most loaded links in the initial network, respectively. The regimes are:

Connectivity driven (Regimen A) *θ*≤*l*_min_—in this case, no links fall below the cost and, hence, no pruning takes place. It is apparent that this regime will essentially have only a fully connected network (the reference network we begin with). Networks in this regime maximize connectivity but their profit is diminished (equation 1).

CP (Regimen B) *l*_min_<*θ*≤*l*_max_—in this regime, the network undergoes the most rapid changes in its structure. All the links that fall below the cost are removed sequentially and the load is redistributed to the remaining network. Nodes gain more traffic and the links that get pruned give rise to a variable CP character. This character is not always prominent in the entire regime and depends strictly on the value of *θ*. An example is shown in Fig. 1b.

Profit-driven (Regimen C) *l*_max_<*θ*—this regime shows extreme structural changes in the network. Most links get pruned except the ones essential for connectivity—eventually giving rise to a tree-like structure towards the end of this regime, illustrated in Fig. 1c. Since we attach the same cost to each link, the cost of the network scales monotonically with the number of links. Thus, networks in this regime have the minimum possible cost.

**Figure 2:**
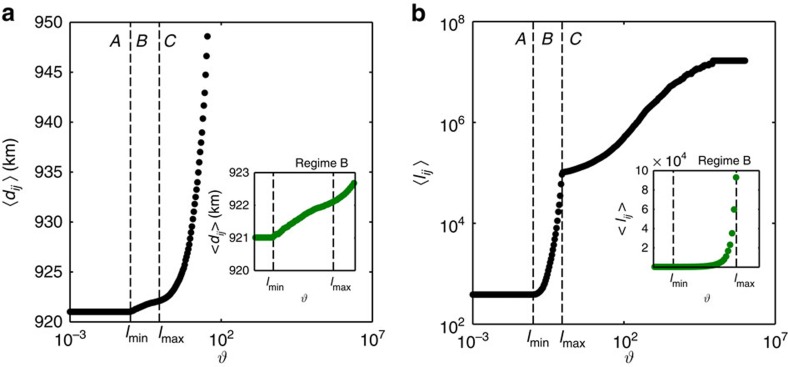
Average shortest path 〈*d*_*ij*_〉 in km and average load 〈*l*_*ij*_〉 dependence on cost *θ*. We observe three different regimes as a function of the cost. In (**a**), the average shortest path length remains relatively stable while the load (a proxy for benefit) as shown in (**b**) increases drastically in regime B. The insets of both figures are blow-ups of regime B. In (**a**), a slight increase in the shortest path in regime B is observed, while in (**b**) the benefit increases by a large magnitude pointing to the inevitable compromise between connectivity and profit. Data are averages over 100 realizations.

Upon removing links and redistributing their loads onto the remaining links, the modularity^18^,^19^, average shortest path length^20^ and average load per link increase; see Fig. 2, while the average degree and average clustering coefficient decrease^20^. This indicates that communities start emerging while keeping beneficial links intact and sacrificing the ones that lead to a shorter path for transfer of loads. The clustering in the network decays due to a periphery that is slowly emerging. It is worth noting that these curves exhibit a kink at *θ*=*l*_max_ (see Fig. 2, Supplementary Note 6 and Supplementary Figs 7–9 for other characteristic properties). With a small increase in the average shortest path (Fig. 2a), the average load on the remaining links increases (Fig. 2b), thereby making them more significant for the network. At *θ*=*l*_min_ the network changes rapidly and links start getting pruned as they fall short of justifying their existence. Around *θ*=*l*_max_, we observe that the network exhausts its pruning capabilities. The links that are removed now are the most loaded and, hence, transfer much more load to other links thereby slowing down the pruning process considerably.

In addition, in regime A, since no link is pruned, the average shortest path length remains constant. As the pruning process becomes effective, the average shortest path slightly increases with the cost (regime B). By contrast, in regime C, the average shortest path increases exponentially with the cost. Note that as illustrated in the Supplementary Figs 10, 11, the fraction of essential links required to ensure global connectivity is small unless the costs are very high, indicating that the constraint of global connectivity does not affect the network's proclivity towards CPs.

### Core size

**Figure 3:**
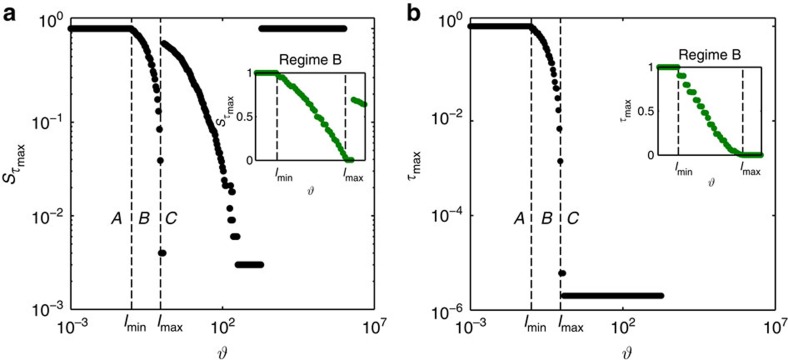
Characteristic metrics of *t*-core decomposition. Core size, 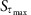, and relative coreness, 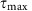, versus the cost, *θ*. (**a**) A decay in the size of the core in regime B for increasing cost is shown. Core size increases again abruptly in the transition between regimes B and C as the pruning mechanism slows down. (**b**) Illuminates upon the comparison of the relative coreness of the core between a fully connected network in regime A and a core–periphery observed in regime B. The insets of both figures are blow-ups of regime B. The core of the network in regime B has a much lower coreness, which decays continuously with increasing cost until the network becomes a tree. Data are averages over 100 realizations.

**Figure 4:**
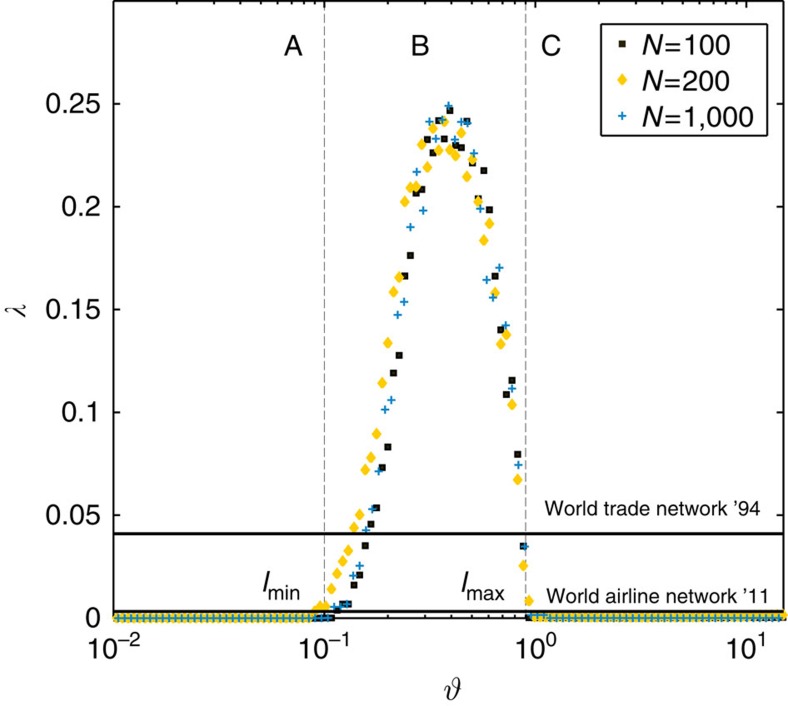
Core–periphery measure *λ* as a function of *θ* for different system sizes *N*. Modelled networks in regime B have a high value of *λ* owing to their core–periphery characteristic and resilience. The World Trade Network from year 1994 lies close to *λ*=0.041 and the World Airline Network from the year 2011 is at *λ*=0.0032. The trade network is only comprised of 80 nodes, whereas, the airline network has close to 3,500 nodes. Data are averages over 100 realizations.

A *t*-core decomposition was performed at every value of *θ* to assess the network's CP properties. We measured the size of the core, 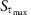, and the maximum relative coreness of the network, 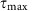, as a function of the cost. Figure 3 shows that in regime A, where the network is still fully connected, the core consists of the entire network with a very large coreness since there are many triangles. On the other hand, in regime C, the tree-like network is sparsely connected such that it is essentially segregated into one shell at coreness, *t*=0. Remarkably, between regimes B and C, the core size exhibits a discontinuity. The network undergoes a transition from a state where the size of the core is comparable to the system size but is of small coreness to a state with a small core and relatively large coreness. Since the empirical WAN is known to have a small core size of ∼2.3% but high inter-connectivity within the core^4^, it should be found in regime B with *l*_min_<*θ*≤*l*_max_, where the value of *λ* is largest (see Fig. 4). *λ* is close to zero in regime A and C, because we have a fully connected network in A and a tree-like one in C. However, in regime B, where *λ*≈0.25 is maximum, we find a periphery emerging which is held together by the core in the middle (see Fig. 1b). In this region, the difference in the relative coreness between core and periphery 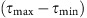 is huge and the ratio of the number of nodes in the periphery to that of the core is much larger than unity 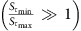. The World Trade Network^21^ and the WAN (ref. 22) are also included in Fig. 4 for comparison (solid horizontal lines). The trade network is only comprised of 80 nodes, whereas, the airline network encompasses about 3,500 nodes. These networks exhibit a lower CP measure, *λ*, since there is a high cost for building networks. In contrast, a network that has no cost (or less cost) attached can comprise many more triangles within its core, consequently depicting a higher value for *λ*. To understand the physical depth of the quantity coreness (*λ*), we first discuss two limits of *λ*: a fully connected network (regime A) and a tree-like structure (regime C). In both cases *λ*=0. We tested another null configuration starting with a fully connected network of our main model where links are removed at random until the network turns into a tree (no more pruning is possible). As shown in Supplementary Fig. 12, by contrast to the results with load redistribution, when links are simply removed at random, there is no well-defined maximum for *λ*, thus CP structures do not emerge at any stage (Supplementary Note 7).

### Coreness distribution

**Figure 5:**
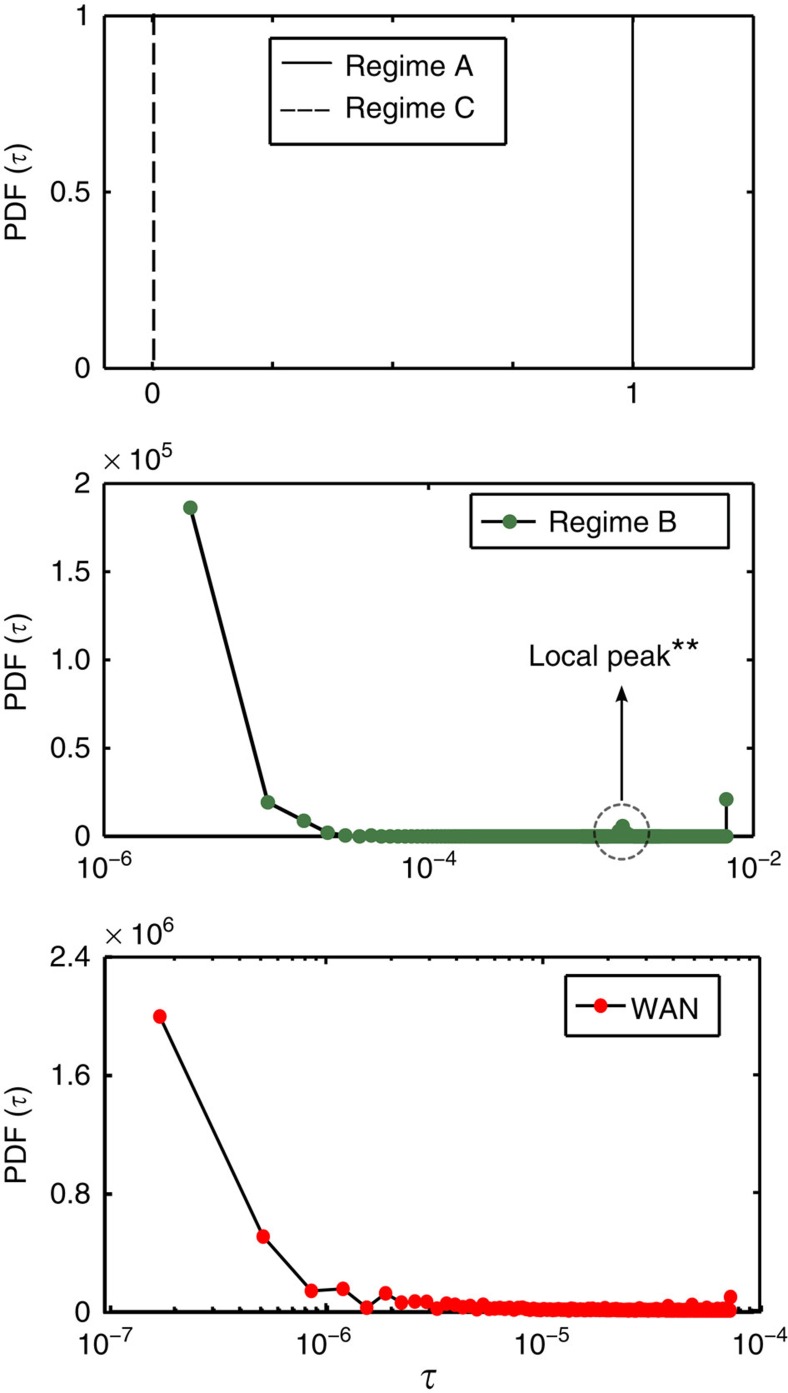
Probability density functions of coreness of different regimes and the empirical WAN. Regime B, for cost *θ*=0.92, that maximizes the value of core–periphery measure (independent of system size *N*), *λ*=0.248 (Fig. 4), and the real-world network exhibit a core–periphery structure. The density functions show the probability of having a shell with relative coreness 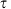 (relative to a fully connected network). Data are averages over 100 realizations.

To evaluate the CP properties of the networks, we calculated the probability density function (PDF) of the relative coreness of some exemplary model networks in each regime as well as the empirical WAN. Figure 5 shows the PDFs of the relative coreness of networks in each regime. Qualitatively, the CP structure is visible in regime B networks. The periphery consists of many nodes with small coreness; probability dropping with increasing coreness (notice the semi-logarithmic scale).

The coreness densities of the networks from regimes A and C exhibit a markedly different behaviour. In the case of a fully connected network (regime A), it consists of a single peak at 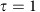 and for the tree-like network (regime C), of a single peak at 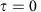. Hence the entire network is segregated into one shell following the t-core decomposition. Because of their simplicity, the PDFs for regimes A and C are grouped in one plot.

Figure 1 illustrates the structural difference between the CP network of regime B and the tree-like network of regime C. It is immediately evident how the core nodes (in black) are highly interconnected as they are grouped closely together by the force directed Fruchterman–Reingold algorithm^23^. The algorithm uses spring-like attractive forces to attract the nodes that have a link between them, while simultaneously repulsive forces of charged particles are used to separate all pairs of nodes. This arrangement allows us to distinguish core from periphery. In the empirical WAN network, the core is spread over continents or different regions of the world (see Supplementary Fig. 13).

### Resilience

Transportation networks in our globalized world have not resulted from a centralized optimization procedure. Most networks have resulted from the superimposition of many locally optimized networks and accretion of regional networks, providing for a globalized way to travel. In such scenarios, it is non-trivial to establish a common ground for measuring resilience. We use a basic measure, often used in the past to qualitatively assess the efficiency of a network^24^ to removal of nodes.

**Figure 6:**
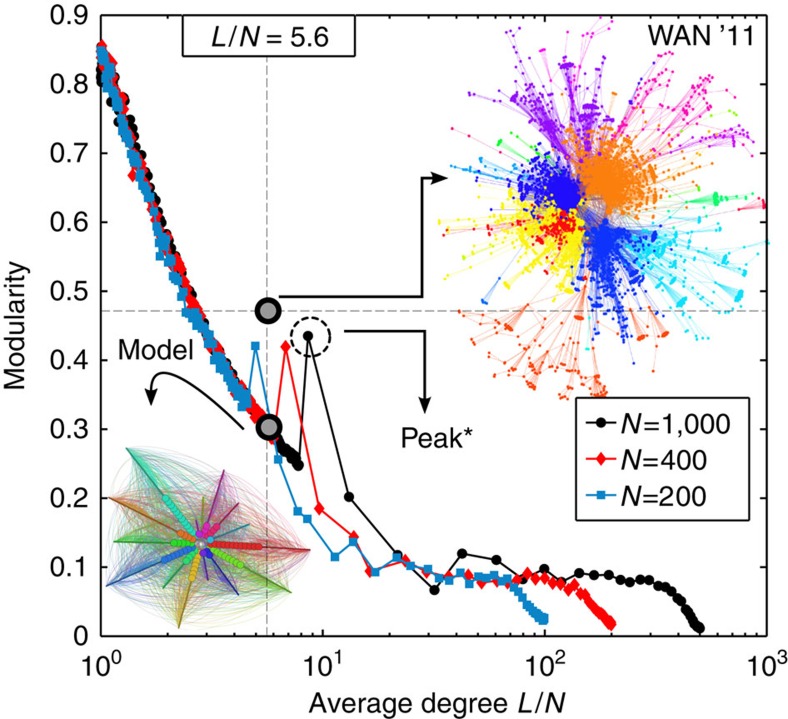
Modularity as a function of average degree. The model networks show a peak* in the modularity for an average degree close to the World Airline Network. This peak is due to the increase in coreness of the network as the core collapses and a larger core takes shape (see Fig. 5—local peak** observed in the distribution of coreness for modelled networks). For the same average degree, *L*/*N*=5.6, the model generates many interconnected modules while the World Airline Network shows little or no links between modules. Different colours represent different communities and the size of the nodes classify them into core (large) or periphery (small). Data for system sizes *N*=200, 400 and 1,000 are averages over 100 realizations.

We compare the robustness of our modelled networks—for the same average degree—with the empirical WAN. As presented in (ref. 4), the empirical network is very sensitive to the removal of high degree nodes and the size of the largest component drops very quickly (Supplementary Note 8 and Supplementary Fig. 14). However, a model network in regime B appears more robust owing its topological strength to a strongly connected periphery where peripheral nodes have a few redundant links between each other. Figure 6 illustrates that the modularity of the network seems to result from the peripheral linkages, a topological feature that indicates the strength of intra-community links over links across communities. This is a grave factor contributing to its abrupt diminishing robustness. Our model produces robust networks that accrue benefit to network elements without compromising on the connectivity of these elements. In addition, the modularity peaks are a result of the increase in coreness of the network as the core collapses and a larger core takes shape (see Fig. 5—local peak** observed in the distribution of coreness for modelled networks). Furthermore, a detailed robustness analysis for various network sizes shows that the change in robustness does not depend on the network size and follows the same pattern for all network sizes (Supplementary Fig. 15). For the same average degree, *L*=*N*=5/6, the model generates many interconnected modules while the WAN shows little or no links between modules (increment in modularity). In other words, the model networks have lower modularity compared with the WAN which also has a larger average shortest path length, giving rise to more tightly knit modules.

## Discussion

We have presented a model producing the qualitative nature of the CP structure observed in many real-world networks. Remarkably, this is possible by dynamically allowing the failed links to redistribute their loads and the network's effort to increase its profit, as two processes working on the network. We have also taken into account the costs imposed due to the spatial nature of such networks, by considering Euclidean distances between the nodes to define the new routes for the redistribution of loads. Simulating these processes on a network with no other fundamental assumptions, we obtain for a wide range of cost values, a small but densely interconnected core and a vast periphery.

Our pruning process not only produces CP networks but also reveals different network regimes. The crossover between these regimes can be modelled using only a single cost-based parameter, *θ*. This parameter can be varied to show many interesting properties of the modelled networks. For instance, when a CP structure is present, the average load on a link (a proxy for the benefit of the link) increases, while the average shortest path length between any two nodes (a proxy for convenience of load transfer) stays stable. In addition, connectivity is optimized in regime A where everything is connected and profit is optimized in regime C, according to the construction of our model. However, note that regime B balances these two real-world considerations and, interestingly, we find most real-world networks to exist in this region as well.

Although, not all networks are planned, their current condition is dictated by a variety of rules. Our efforts do not reproduce every kind of network verbatim and do not try to fully describe the evolutionary process of a network but give a plausible explanation for understanding profit-driven CP networks. We not only produce the CP character of networks but also show that modelled networks are more resilient to removal of nodes compared with the empirical example of the WAN. This resilience can be attributed to the less modular structure of the modelled networks. Since our modelled networks are stable and resilient to removal of nodes, it is natural to ask whether our approach could be used to design cost-efficient and resilient infrastructure networks, something policy makers might centrally control.

The process of pruning a globally connected network fundamentally differs from the bottom-up growth many real networks have undergone. Schneider *et al.*^25^ developed a pruning model, which reproduces well many topological properties of protein interaction networks. Inspired by this strategy of preferential depletion, our model mimics CP networks closely. Transport networks with a geographical dependence try to optimize faster connectivity with demand induced profit. An example includes the WAN that is a possible outcome of individual airline networks competing and cooperating (wherever profitable) with each other. On the other hand, the networks of large carriers like Star Alliance could approximate the picture of a global network in which our model could make suggestions for improvements assuming the partners in such an alliance are able and willing to cooperate with each other.

Lastly, Peixoto *et al.*^26^ show that the most robust topology against random failures is a CP structure. By studying the percolation properties of arbitrary large-scale networks using robustness as the most significant force for driving the system, the authors show that a CP network is the case of maximum entropy. Our non-equilibrium approach depicts that a network in regime B (critical window) will be highly robust in comparison to real networks. Louf *et al.*^27^ have proposed a cost-benefit driven optimization model based on physical distances in transport networks to study their formation. An interesting revelation of their work is that cost driven network optimization leads to a hub-and-spoke structure, different from a CP structure in our model. Louf *et al.* carried out the addition of links on a static system where the distances dictate the future of links. Our model differs from this in a way that a dynamic redistribution of loads is taken into account, which encapsulates the collective nonlinear effects of various local load redistributions around the network. The interplay between load redistribution and profit provides a plausible explanation for CP in transport networks. We believe that our framework can be extended to other networks that are based on profit maximization.

## Methods

### Sample size

We ran tests for various system sizes, namely, *N*=100, 200, 400,… 1,000 and for each system 100 randomly selected samples were considered.

## Additional information

**How to cite this article:** Verma, T. *et al.* Emergence of core-peripheries in networks. *Nat. Commun.* 7:10441 doi: 10.1038/ncomms10441 (2016).

## Supplementary Material

Supplementary Information Supplementary Figures 1-23, Supplementary Tables 1-6 and Supplementary Reference.

Supplementary Movie 1 An animated illustration of the relationship between popularity of a node and its load. As the cost of the system, ϑ, increases, this relationship starts breaking. The color map shows the coreness of nodes in the network and at each cost step the nodes change color. In the beginning in regime A the nodes are all of the same color indicating that they have the same coreness. As the cost increases, the high coreness nodes start to appear at nodes that have a high initial popularity; the ones forming hubs for the core. After a certain cost when the network is close to the end of the critical window, there are only two colors that appear for two different layers of coreness showing that a bigger core encapsulated the inner core to break this characteristic feature of the network. In regime C, the network shows only one color (layer) indicating the start of the tree-like regime.
