# Supplementary material for: Emergence of core–peripheries in networks
Source: Nat Commun. 2016 Jan 29;7:10441. doi: 10.1038/ncomms10441 (PMC4740181; doi:10.1038/ncomms10441)
Supplement: Supplementary Information — Supplementary Figures 1-23, Supplementary Tables 1-6 and Supplementary Reference. [file ncomms10441-s1.pdf]

## Supplementary Figures

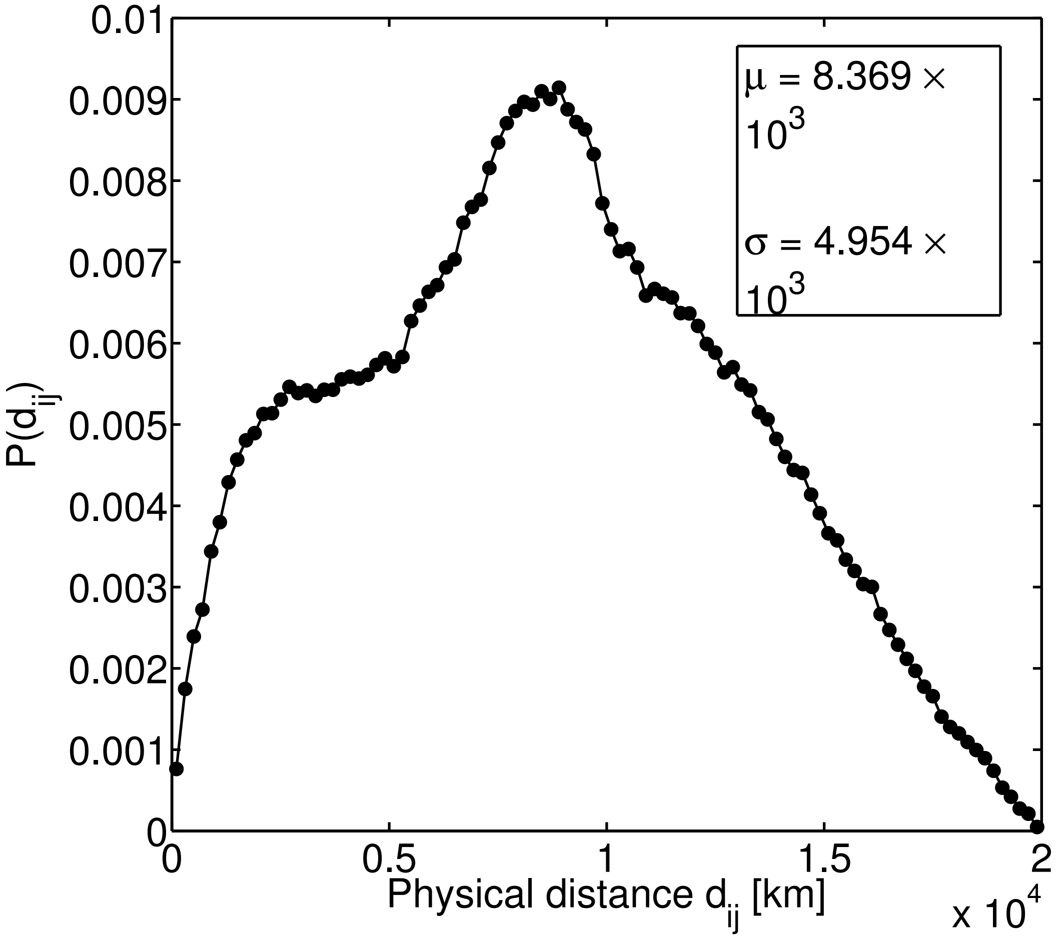


Supplementary Figure 1: **Distribution of physical distances** $d_{ij}$ between nodes in the initial state of the model network.

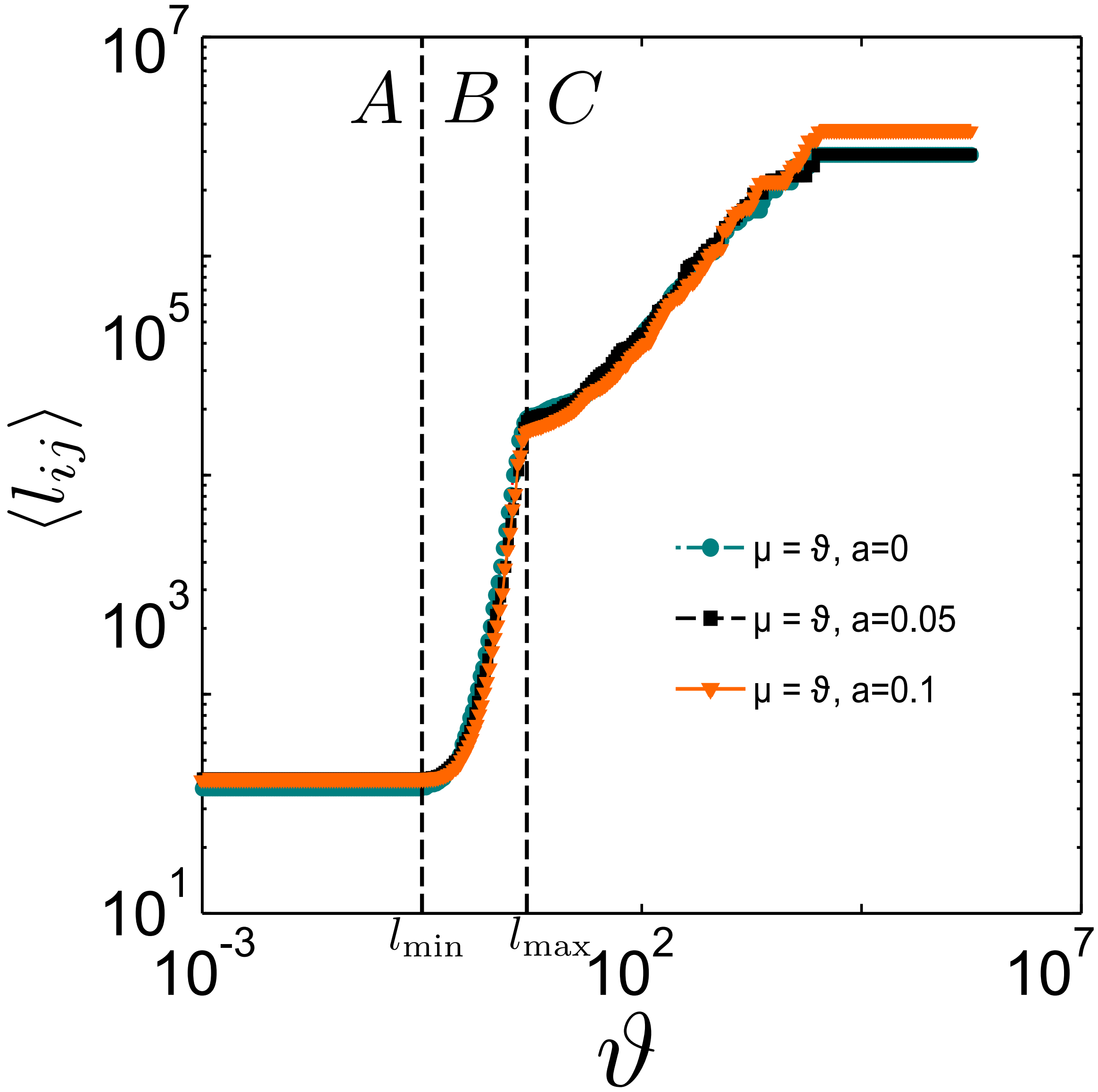


Supplementary Figure 2: **Average load $\langle l_{ij} \rangle$ dependence on cost $\vartheta$ for varying dispersion in the value of the cost threshold.** We observe three different regimes as a function of the cost. The effect of increasing the cost systematically is robust against small changes in dispersion of the cost itself. We produce heterogeneity for the threshold parameter, $\vartheta = c_{ij}$; $c_{ij} = (1+\delta_{ij})c$, where $\delta_{ij}$ is a uniformly distributed random number in the range $[-a;a]$. $\mu$ depicts the mean of the varying cost. The dispersion - in particular, we consider the cases $a = \{0, 0.05, 0.1\}$ - produces three different scenarios. The load (a proxy for profit) increases drastically in regime B showcasing a core-periphery network's existence. Data are averages over 100 realizations.

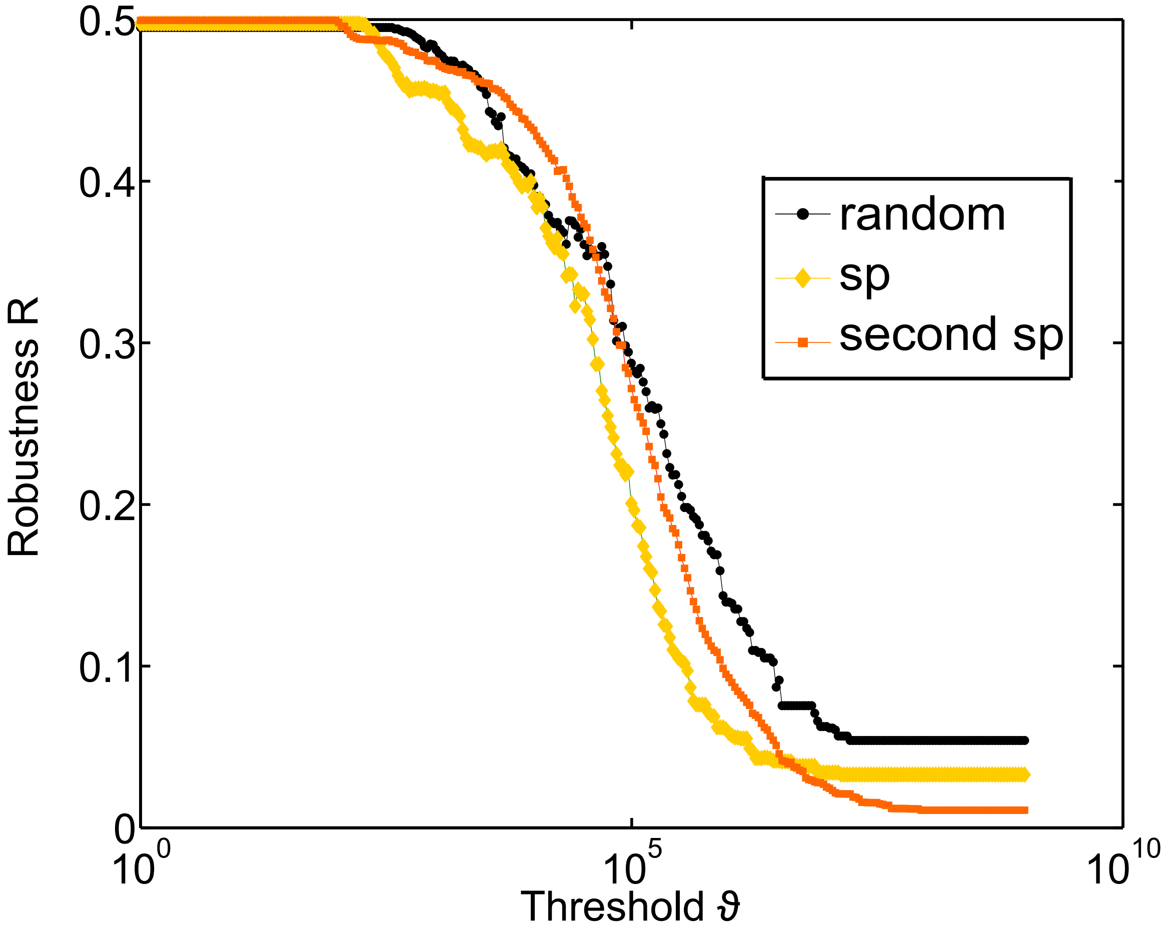


Supplementary Figure 3: **Change in robustness, $R$, vs threshold, $\vartheta$.** The change in robustness as load is redistributed follows the same pattern as threshold is increased for three different scenarios of path selection. The first case is random selection wherein paths are selected randomly for redistributing load. The second case is the standard for our model; selecting shortest path for redistribution. The third case incorporated redistribution of load over the second shortest path. Data are averages over 100 realizations.

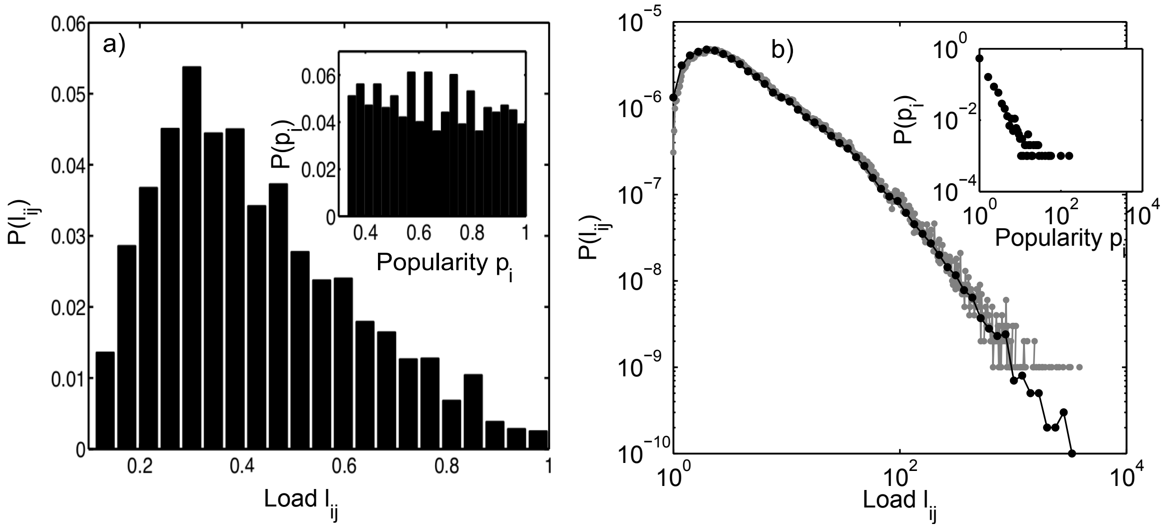


Supplementary Figure 4: **Initial distribution of loads on each link**. Inset of the figure shows the initial popularity assigned to the nodes, chosen from an underlying distribution. Figure a) depicts uniform popularity (no knowledge of hubs) randomly assigned from the interval, $[0.33, 1]$ and Figure b) shows a power law distribution, $P(p) \sim k^{-\gamma}$, with $\gamma = 2.5$ depicting the presence of hubs. The black smooth curve is a fit of large bin sizes, the grey curve shows unbinned data. Data are averages over 100 realizations.

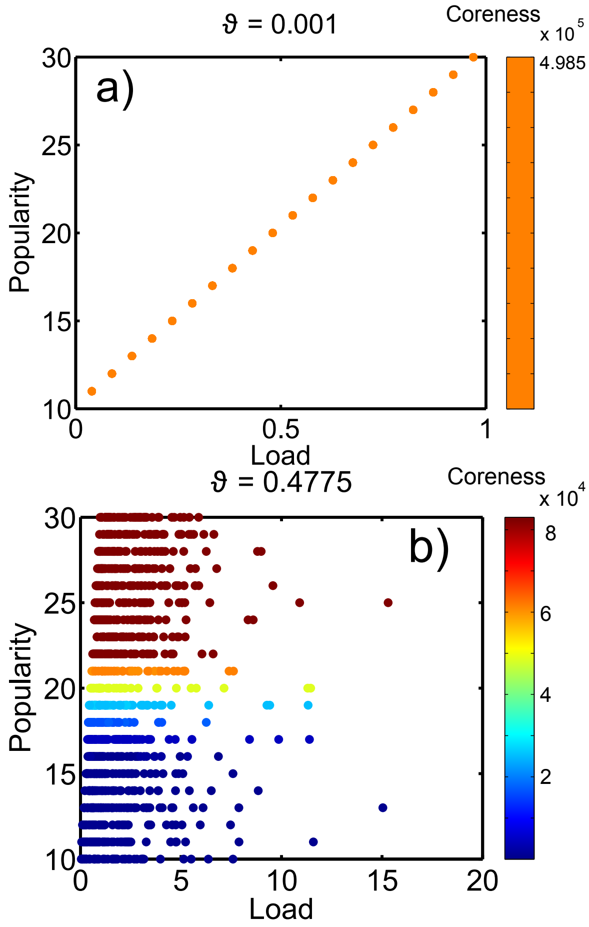


Supplementary Figure 5: **Distribution of loads per node vs initial popularity of nodes**. Figure a) exhibits the relationship between initial popularity and load in regime A and Figure b) shows the correlation between popularity and loads in regime B. It is evident that the higher popularity nodes at the beginning tend to form the core towards the end. A step-varying animation of this relationship over the duration of change in cost shows a uniform distribution in the beginning (initial conditions) that later transforms into higher popularity nodes forming the core and then breaking it down to transition into a tree-like network in regime C (see Supplementary Movie).

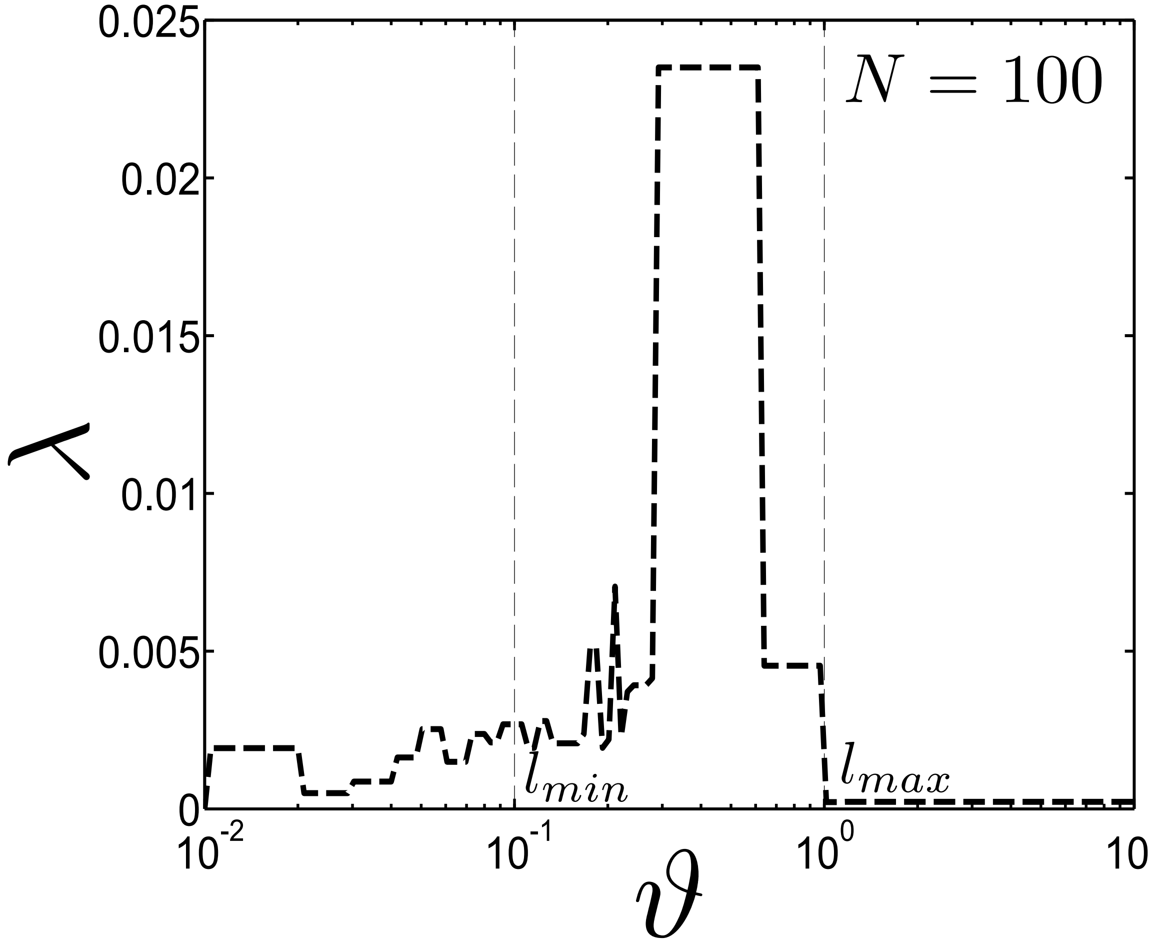


Supplementary Figure 6: **Core-periphery measure $\lambda$ as a function of $\vartheta$ for a system of $N = 100$ nodes**. Modeled networks in the critical window (B) have a high value of $\lambda$ owing to their core-periphery characteristic and resilience. There is no typical transition at $l_{min}$ as the links are removed from the beginning and the same characteristic transition at $l_{max}$ is observed, as we do for a different load choice. This model is based on betweenness as the sole choice of load indicating that our model illustrates the basic ingredients of a core-periphery network irrespective of the initial conditions and choice of link loads. Data are based on 100 realizations.

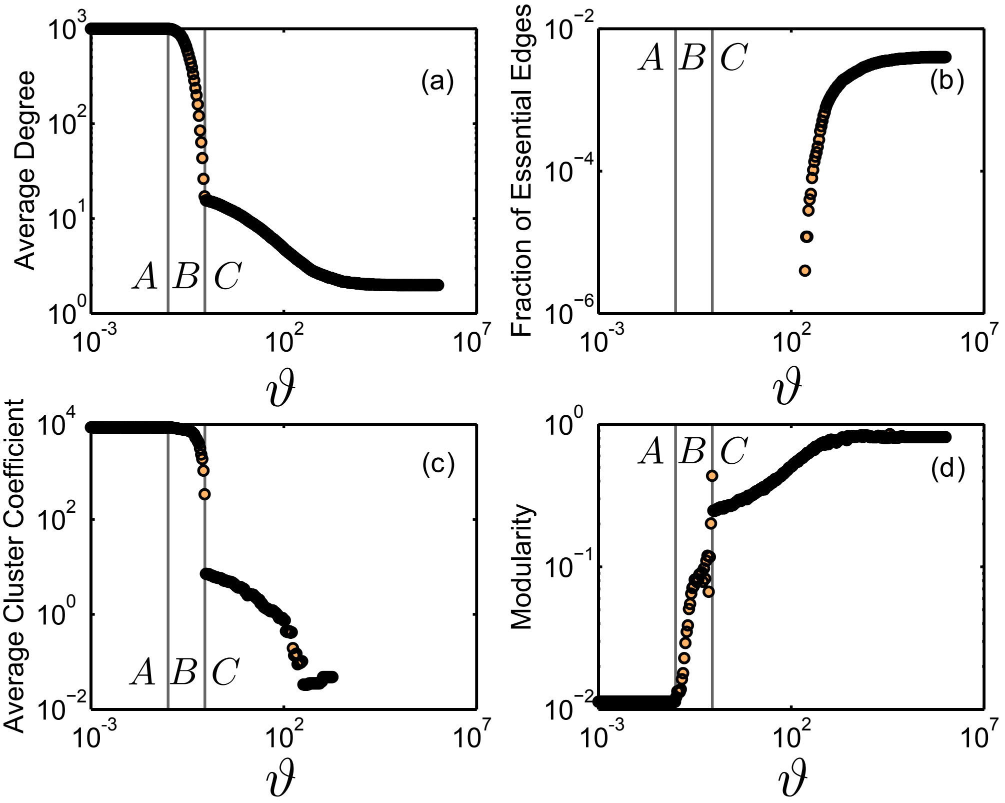


Supplementary Figure 7: **Network properties measure with respect to a varying cost,** $\vartheta$. (a) shows the relatively abrupt drop in average degree. (b) Fraction of essential edges that need to be there to maintain a connected skeleton of the system. (c) Average clustering coefficient drops in regime B. (d) Modularity increases as communities start appearing. Data are averages over 100 realizations.

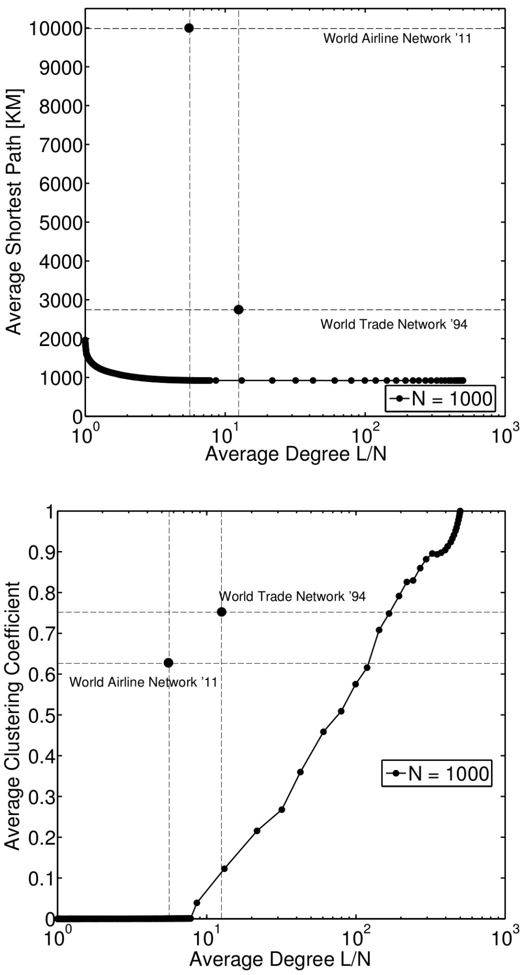


Supplementary Figure 8: **Comparison of the real world network data with our model networks for same average degree**. The empirical networks have a higher clustering coefficient and much longer paths on average to transport load. Data are averages over 100 realizations.

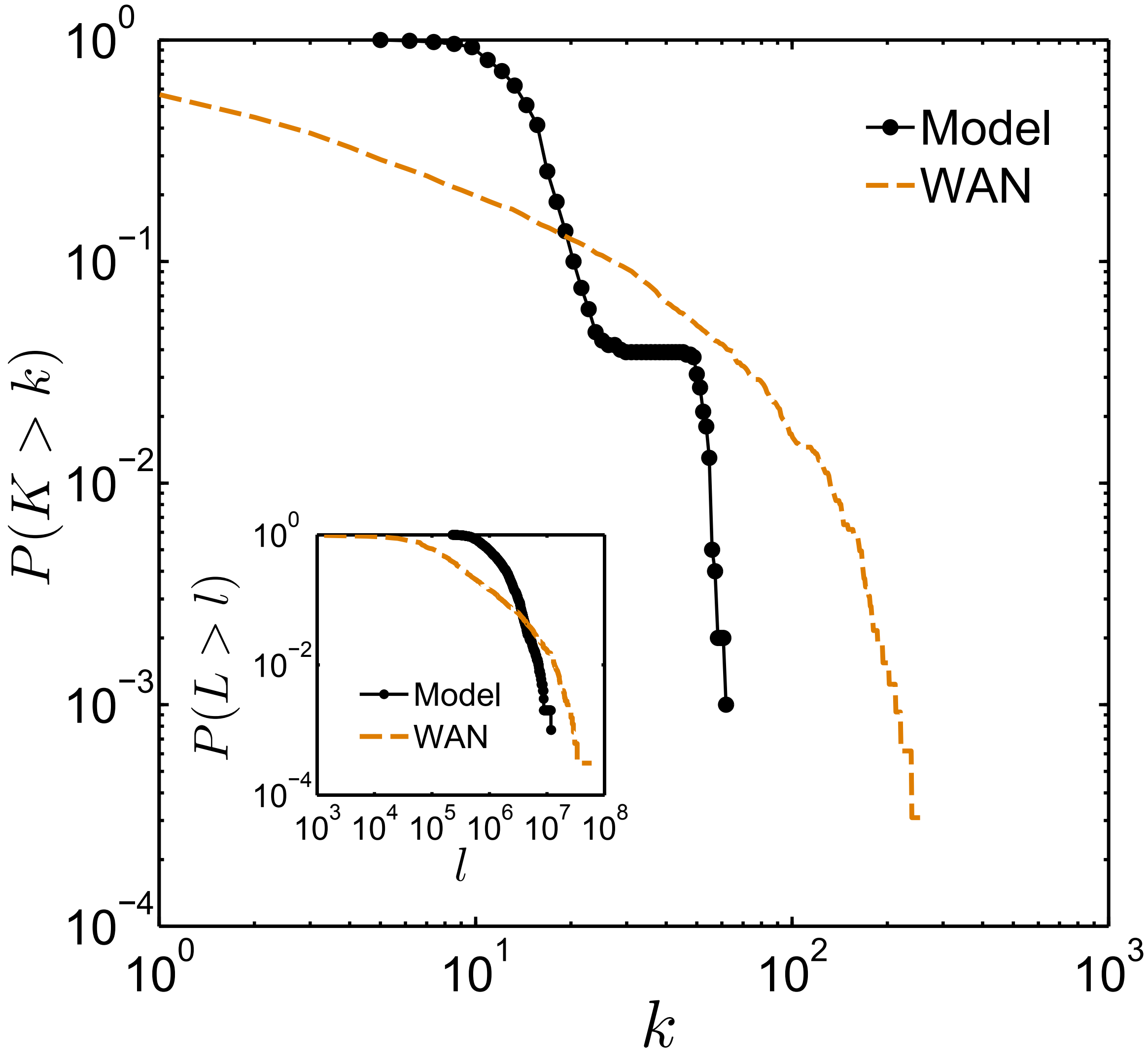


Supplementary Figure 9: **Degree distributions, $P(K > k)$, of a model network in regime B and the world airline network**. Inset of the figure shows the load distributions, $P(L > l)$, of a model network in regime B and the world airline network. The model networks lack a characteristic scale-free property which is clear and present in most real-world networks of the sort due to existence of hubs.

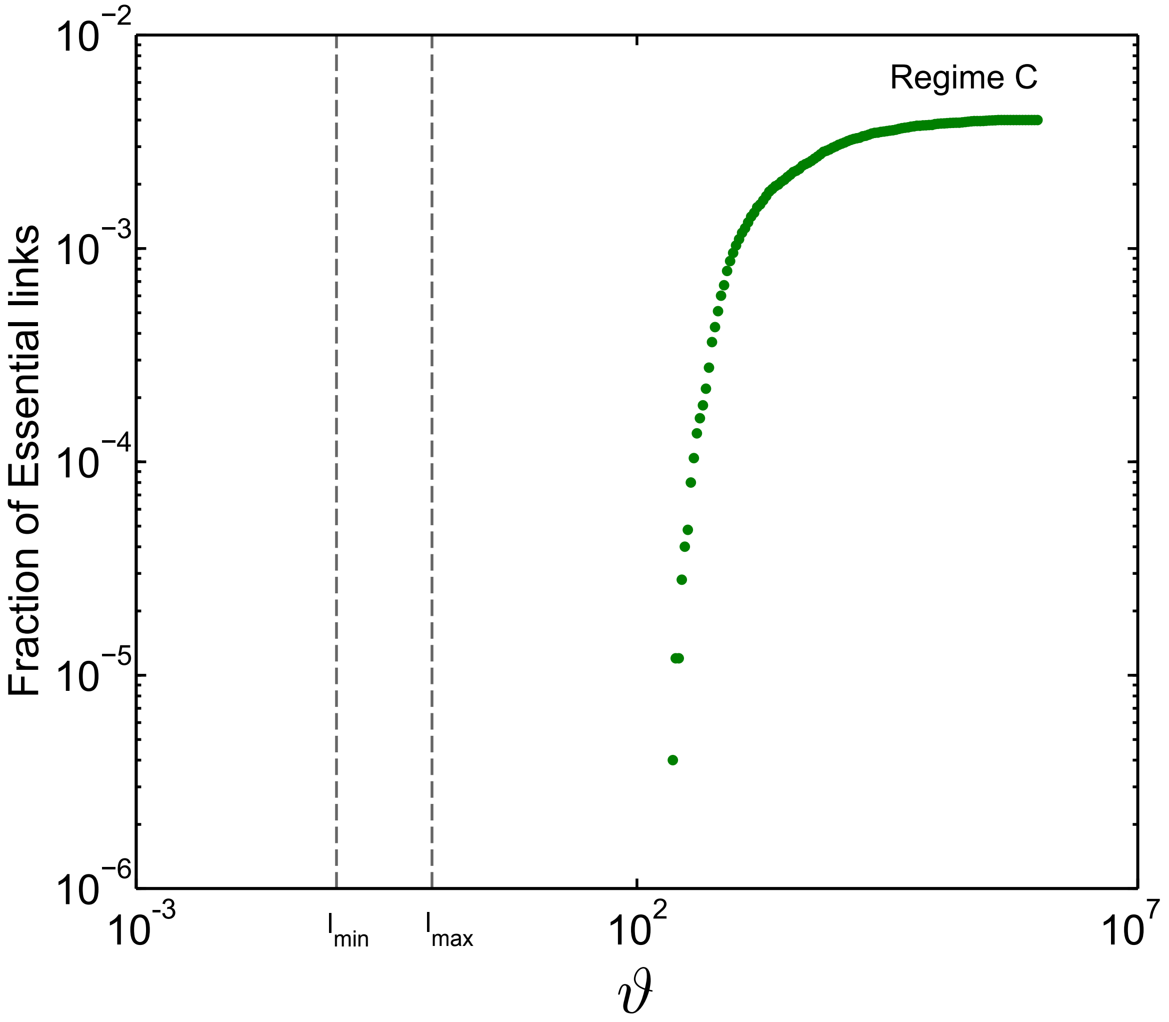


Supplementary Figure 10: **Fraction of essential links vs $\vartheta$.** The fraction of links that are essential to maintain connectivity only increases in the profit driven regime (C). Before that it remains zero suggesting that the assumption of maintaining connectivity in the system does not give rise to core-peripheries. Data are averages over 100 realizations.

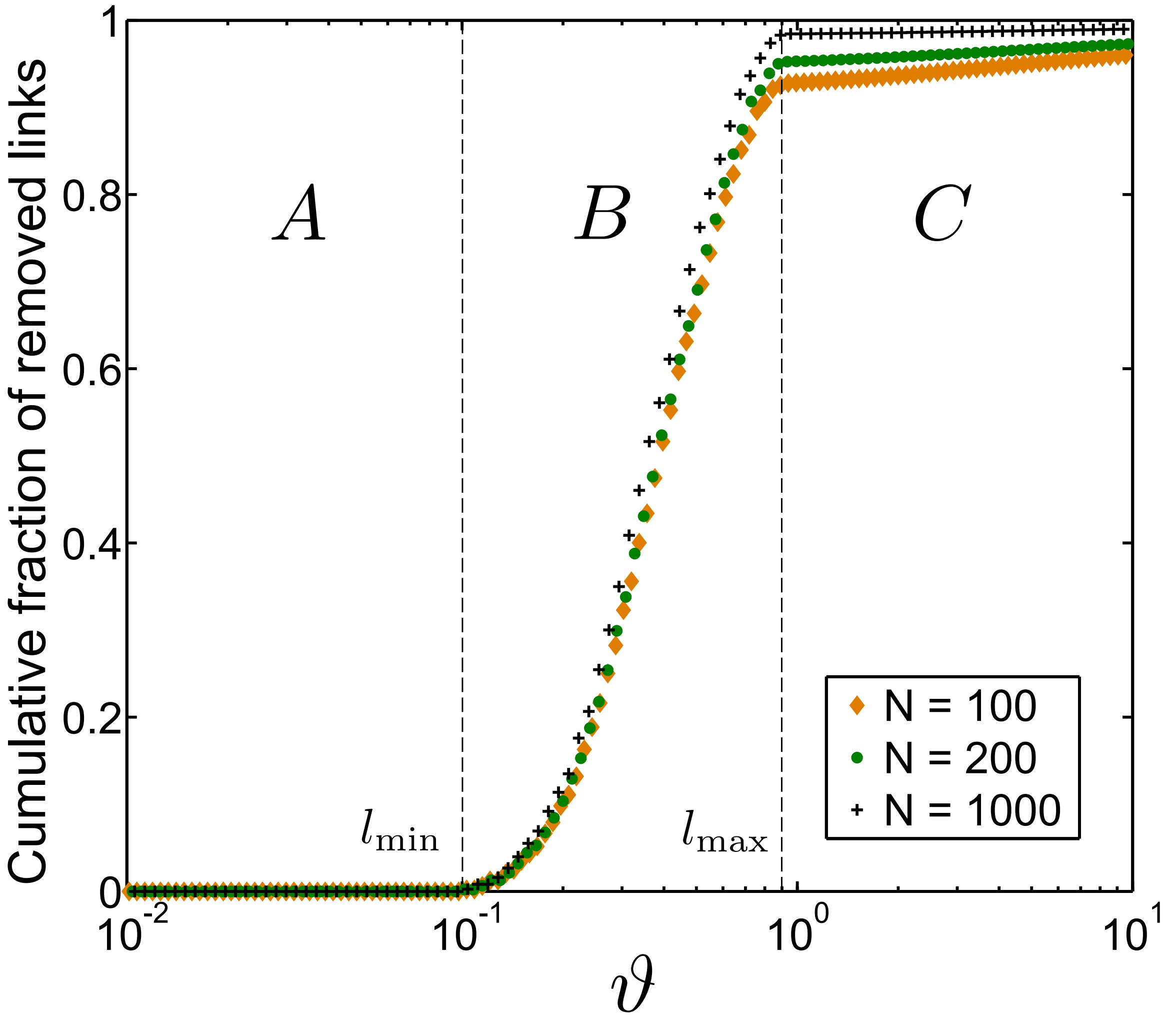


Supplementary Figure 11: **Fraction of removed links as a function of $\vartheta$ for different system sizes** $N$. The critical window (B) exhibits the largest change in the removal of links from the network, irrespective of the system size. The network undergoes a substantial change when $l_{\min} < \vartheta \leq l_{\max}$. Data are averages over 100 realizations.

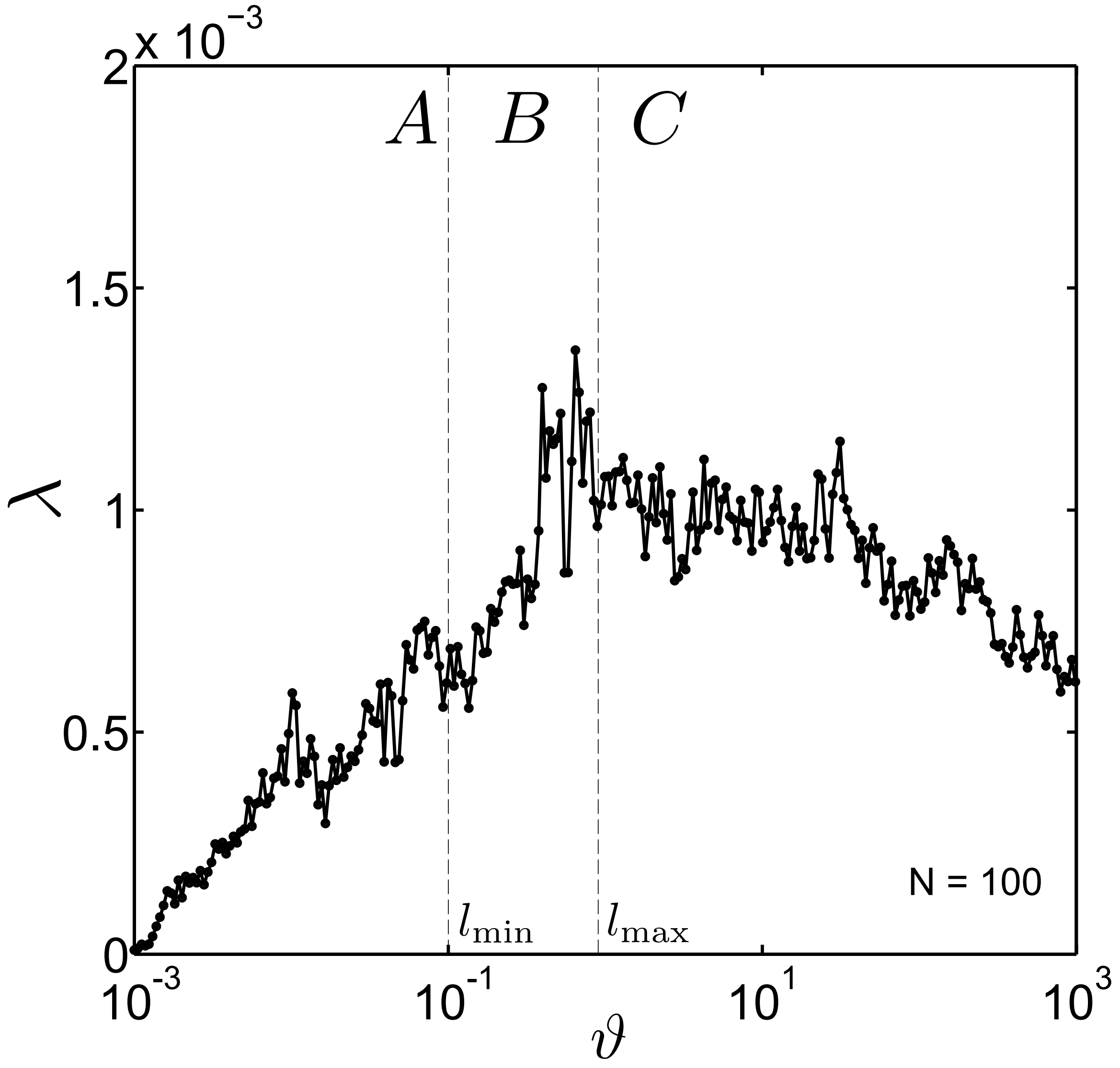


Supplementary Figure 12: **Core-periphery measure $\lambda$ as a function of $\vartheta$.** The links are removed at random for a system of $N = 100$ nodes. All networks have a varying value of $\lambda$ without indicating a pronounced peak or preferable region for a core-periphery network (see Supplementary Note 5 - Supplementary Figure 6). Data are based on 100 realizations.

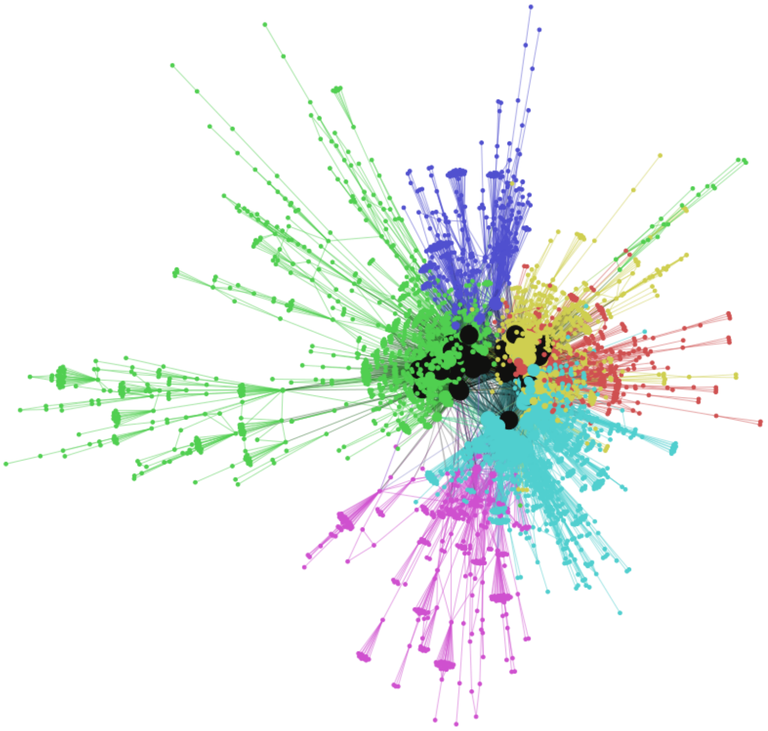

Supplementary Figure 13: **Visualization of the World Airline Network ($N = 3237$).** The node layout was generated by applying the Fruchterman-Reingold algorithm [2]. Size of the nodes show the difference in magnitude of coreness with the largest (black) indicating the core and the smaller different colors - the periphery - showing different continents.

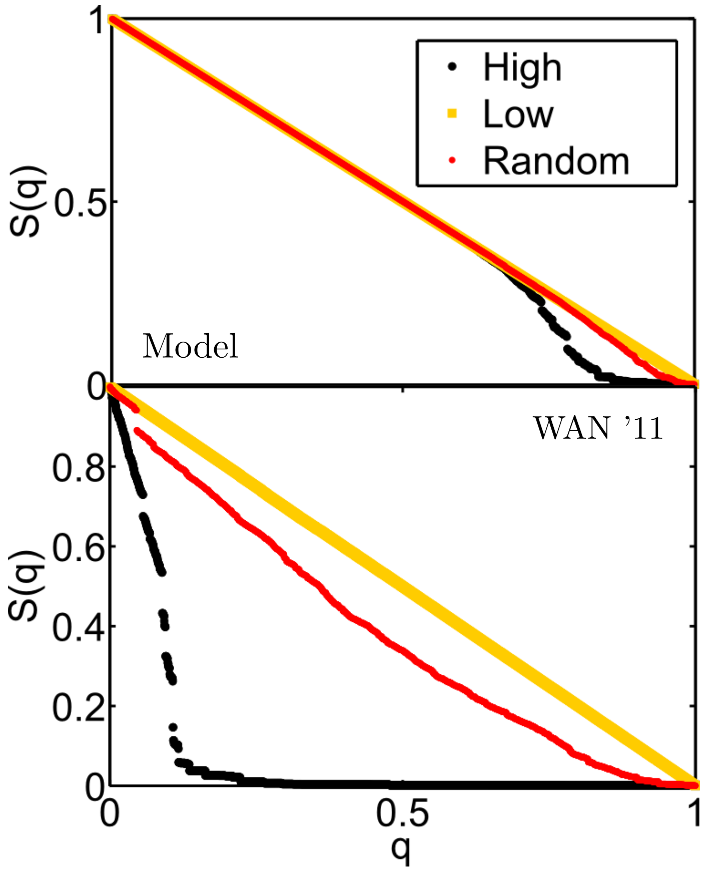


Supplementary Figure 14: **Connectivity robustness curves for networks belonging to different regimes**, showing the fraction of nodes in the largest connected component of the network as a function of the fraction of nodes that have been iteratively removed. The three colors represent removal strategies: starting with the highest degree (black), lowest degree (yellow) or in a random order (red). Data are averages over 100 realizations.

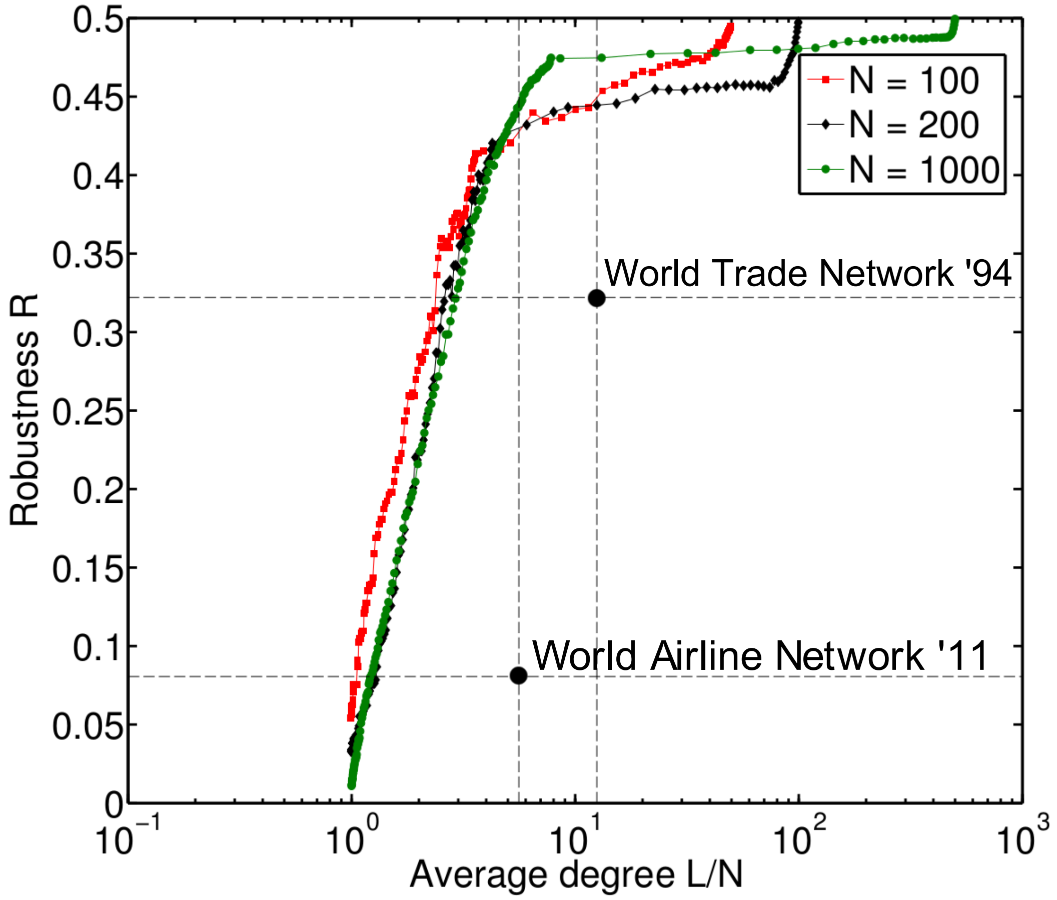


Supplementary Figure 15: **Robustness versus average degree**. The model networks show the same robustness pattern for changing average degree. Data from the real-world networks, WAN and WTN is provided as evidence to show the robustness improvement of model networks. Data for system sizes $N = 100, 200, 1000$ are averages over 100 realizations.

# Supplementary Notes

## Supplementary Note 1 - Distance Distribution

We do not take into account the exact spatial positions of the nodes. The nodes are distributed randomly on a sphere of the size of the Earth's radius. A link is characterized by its physical length $d_{ij}$ (distance between nodes, in km, taken randomly from a Gaussian distribution, $\mu = 8.369 \times 10^3; \sigma = 4.954 \times 10^3$. Supplementary Figure 1 shows the probability distribution of the physical distances between nodes spread around the globe, in kilometers, for a weighted analyses.

## Supplementary Note 2 - Cost Variations

We have run different sets of simulations with $c_{ij} = (1 + \delta_{ij})c$, where $\delta_{ij}$ is a uniformly distributed random number in the range $[-a; a]$. In particular, we consider the cases $a = \{0, 0.05, 0.1\}$. As seen in Supplementary Figure 2, we obtain good quantitative agreement for the three cases, showing that our results are robust to heterogeneity in the parameter $c_{ij}$.

## Supplementary Note 3 - Popularity

To each node in the network, we randomly assigned a popularity from a uniform distribution in one case and a scale-free distribution, in another. This popularity corresponds to the relative relevance of a node. Supplementary Figure 4 depicts the load assigned to each link using the rule, $l_{ij} = p_i p_j$, where $p_i \in [0.33, 1]$ for the uniform distribution and $P(p) \sim k^{-\gamma}$, with $\gamma = 2.5$ for the power-law distribution. Small values are eliminated for simplifying numeric calculations. The behavior of average load and average distance remains qualitatively identical with changes in threshold, $\vartheta$, for both cases.

## Supplementary Note 4 - Popularity versus Load

Supplementary Figure 5 illustrates that as the cost, $\vartheta$, increases, the correlation between initial popularity and load weakens. Initially the nodes are part of the same core as it is a fully-connected network. With increasing cost, the nodes are segregated into different cores. In the beginning, in regime A the nodes are all of the same color indicating that they have the same coreness. As the cost increases, the coreness of the nodes with a high initial popularity raises, and thus they become the hubs. After a certain cost when the network is close to the end of the critical window, there are only two colors that appear forming two different layers of coreness showing that a bigger core encapsulates the inner core to break this characteristic feature of the network. In regime C, the network shows only one color (layer) indicating the start of the tree-like regime.

The redistribution mechanism changes the load passing through nodes by increasing the network traffic for certain nodes, thereby creating hubs that give rise to the core-periphery nature of the network (see Supplementary Movie).

## Supplementary Note 5 - Load

The choice of load for our model is specific in the sense that the least loaded link is removed at every iteration and this load is determined from redistribution. This case is essentially a generalization of the more conventional load choice, betweenness centrality, because in this case when a link is removed, the betweenness of other links is increased as load is redistributed to other shortest paths. At each iteration we recursively rank the links according to betweenness centrality and remove the one with the lowest betweenness. After the removal we recalculate the betweenness centrality of each link. Note that, shortest paths previously going through a link are rerouted when the link is removed, resembling the redistribution mechanism described

above. To include disorder into the distribution of betweenness, we consider a weighted betweenness $B_{ij}$ of the link $ij$, defined as:

$$B_{ij} = \sum_{i \neq j \neq s \neq t} \frac{n_{st}^{ij} W_{st}}{n_{st}}, \quad (1)$$

where $n_{st}$ is the total number of shortest paths connecting nodes $s$ and $t$, $n_{st}^{ij}$ is the subset of such paths containing the link $ij$, and $W_{st}$ is the weight of the pair $st$ that we set randomly from a uniform distribution in the interval $[0.5; 1.5]$. As shown in Supplementary Figure 6, for this pruning process we also obtain a peak in $\lambda$, in the window spanning $l_{min}$ and $l_{max}$, corresponding to a core-periphery structure. This clearly supports that our results are robust to the choice of load.

We run simulations with two other path alternatives for load redistribution. Firstly, a path is chosen randomly for redistribution of load. In the second scenario, the load is redistributed over the second shortest path available. These two scenarios are contrasted with our standard shortest path scenario, depicting that the robustness results - with a varying cost threshold - in all cases follows the same pattern (see Supplementary Figure 3).

## Supplementary Note 6 - Topological characteristics of the real world

The empirical networks have a higher clustering coefficient and much longer paths on average (Supplementary Figure 8), likely due to geographical restrictions. The degree and load distributions show that our model lacks a scale-free nature (Supplementary Figure 9) which is more clearly visible in the real-systems due to the existence of hubs.

## Supplementary Note 7 - Coreness

In order to understand the physical depth of the quantity coreness, $\lambda$, we discuss two limits of $\lambda$: a fully connected network (regime A) and a tree-like structure (regime C). In both cases $\lambda = 0$. Then, we consider a null-model. We have taken a fully-connected network and removed links at random until it turns into a tree (no more pruning is possible). As shown in Supplementary Figure 12, by contrast to the results with load redistribution, when links are simply removed at random, there is no pronounced maximum for $\lambda$, thus core-periphery structures do not emerge at any stage.

## Supplementary Note 8 - Resilience

As an additional method of comparing modeled networks to the real network, we use a connectivity robustness measure as defined in Ref. [1]. For a given network, this scheme assesses how robust the connectivity of the largest connected component is against the removal of nodes or links. The following iterative steps are taken when removing nodes, with a), b) and c) denoting three separate versions of the removal procedure:

- Create a list of nodes ordered by their degree.
- Remove the node with the a) maximum degree, b) minimum degree or c) a random node.
- Measure the size (relative to the system size $N$) of the largest connected component $S(q)$ as a function of the fraction of removed nodes $q$ and repeat until all nodes have been removed.

We performed a finite-size study of the results in modularity. We considered $N = \{100, 200, 400, 600, 800, 1000\}$ but only three different sizes are shown in Supplementary Figure 15 (for the sake of clarity). We compare the robustness curves of a modeled network of the same average degree with that of the empirical world airline network. A detailed robustness analysis collapse for various network sizes shows that the change in

robustness does not depend on the network size and follows the same pattern for all network sizes, as now shown in Supplementary Figure 15.

# Supplementary Methods

## Load Redistribution

*Algorithm.* We start the simulations with a fully connected network and follow the steps sequentially,

1. Create an ordered list of passenger loads, $l_{ij}$, and choose the link with the smallest load $l_{ij}^{\min}$. If there are several links with an equally small load, choose one randomly.

2. Say $A_{ij}$ represents the potential link between nodes $i$ and $j$ and can take values 1 or 0 depending on whether the link is present or not, respectively. If the load of the chosen link falls below the cost, $\vartheta$, and if it has not been labeled essential (as defined in 3(a).), delete the corresponding link as follows, if $l_{ij}^{\min} < \vartheta \rightarrow A_{ij} = A_{ji} = 0$.

3. Check whether after removing this link the network is still connected (single component).

   (a) If it becomes disconnected, reverse the removal and mark this link as "essential" such that it does not get selected for removal again.

   (b) Else, find the new shortest path between $i$ and $j$ (e.g. Dijkstra's algorithm [3]), given that their direct link has been deleted. When finding the shortest path, use the Euclidean distances $d_{ij}$ as link weights (refer to section Model for more details).

4. Reroute the passenger load of the deleted link to each link that is part of the new shortest path. For example, if the shortest path between $i$ and $j$, $SP(i,j)$, passes through a set of nodes $K$, set,

$$\begin{aligned} &\text{for each link } (k,k') \in SP(i,j) \\ &l'_{kk'} = l_{kk'} + l_{ij}^{\min} \end{aligned} \tag{2}$$

5. Mark $l_{ij}^{\min}$ as "removed" and repeat the above steps until only essential links fall below the cost, $\vartheta$.

# Supplementary References


[1] Verma, T., Araújo, N. A., Herrmann, H. J. Revealing the structure of the world airline network. *Sci Rep* **4**, 5495 (2014).

[2] Fruchterman, T. M. J., Reingold, E. M. Graph drawing by force-directed placement. *Software: Practice and Experience* **21**, 1129–1164 (1991).

[3] Dijkstra, E. W. A note on two problems in connexion with graphs. *Numerische mathematik* **1**, 269–271 (1959).
